# Optimization of Geometry Parameters of Inkjet-Printed Silver Nanoparticle Traces on PDMS Substrates Using Response Surface Methodology

**DOI:** 10.3390/ma12203329

**Published:** 2019-10-12

**Authors:** Jumana Abu-Khalaf, Loiy Al-Ghussain, Ahmad Nadi, Razan Saraireh, Abdulrahman Rabayah, Safwan Altarazi, Ala’aldeen Al-Halhouli

**Affiliations:** 1 Department of Mechatronics Engineering/NanoLab, School of Applied Technical Sciences, German Jordanian University, Amman 11180, Jordan; jumana.abukhalaf@gju.edu.jo (J.A.-K.); loiy.al-ghussain@uky.edu (L.A.-G.); a.nadi@gju.edu.jo (A.N.); sarayrah99@gmail.com (R.S.); a.rabayah@gju.edu.jo (A.R.); 2 Mechanical Engineering Department, University of Kentucky, Lexington, KY 40506, USA; 3 Department of Industrial Engineering, School of Applied Technical Sciences, German Jordanian University, Amman 11180, Jordan; safwan.altarazi@gju.edu.jo; 4 Institute of Microtechnology, Technische Universität Braunschweig, 38124 Braunschweig, Germany; 5 Faculty of Engineering, Middle East University, Amman 11831, Jordan

**Keywords:** inkjet printing, silver nanoparticles, PDMS, stretchable circuits, response surface methodology, optimization

## Abstract

Inkjet printing is an emerging technology with key advantages that make it suitable for the fabrication of stretchable circuits. Specifically, this process is cost-effective and less complex compared to conventional fabrication technologies. Inkjet printing has several process and geometry parameters that significantly affect the electromechanical properties of the printed circuits. This study aims to optimize the geometry parameters of inkjet-printed silver nanoparticle traces on plasma-treated polydimethylsiloxane (PDMS) substrates. The optimization process was conducted for two printed shapes, namely straight line and horseshoe patterns. The examined input factors for the straight line traces were: the number of inkjet-printed layers and line width. On the other hand, the number of cycles and amplitude were the examined input parameters for the horseshoe shape. First, the optimal number of layers and line width were found from the straight line analysis and subsequently were used in the optimization of the horseshoe pattern parameters. The optimization of the input parameters was carried out using the response surface methodology (RSM), where the objective of the optimization was to maximize the breakdown strain of the traces while maximizing the gauge factor and minimizing the ink cost. The results indicate that a 1.78 mm line width and one layer are the optimal geometry parameters for the straight line traces, while for the horseshoe pattern, the optimal parameters are one layer, a line width of 1.78 mm, amplitude of 4 mm and one cycle. The optimal straight line was designed to sustain up to 10% strain while the horseshoe pattern was designed to sustain up to 15% strain.

## 1. Introduction

In the last decade, printed electronics (PE) have been extensively employed in many areas such as biomedical and automotive systems, robotics, and military and healthcare sectors [1,2,3]. Several technologies that can be used for the fabrication of PE can be found in the literature. These include screen printing [4,5,6,7], photolithography [8,9] and inkjet printing [10,11,12,13]. Compared to screen printing, photolithography and other fabrication technologies, inkjet printing is considered a low-cost, contactless, fabrication technique that requires less complex fabrication steps. Hence, inkjet printing has been utilized extensively in the fabrication of PE. In addition, inkjet printing technology has the capability of depositing a wide range of conductive inks on PE substrates, where most of these inks contain suspended nanoparticles such as silver, carbon, gold and copper [1,14,15]. Various materials have been used as the base substrate of PE such as silicon wafers [16,17], polyimide [18,19,20], and polydimethylsiloxane (PDMS) [4,8,17,21,22,23,24,25], where the base material plays a vital role in determining the PE mechanical characteristics [10]. For instance, the use of PDMS as a base material grants PE with the stretchability characteristics required to provide skin-like and light-weight characteristics that enable their use in many novel applications [26].

Stretchable circuits are one of the emerging examples of PE. They are being utilized in vast applications, especially in the biomedical field, due to their desirable characteristics. The electromechanical properties of stretchable circuits, namely the breakdown strain and strain gauge, are vital. In the literature, numerous research studies have investigated the optimization of these properties extensively [7,23,27,28,29,30]. For instance, inkjet printing technology has several process parameters that significantly affect the electrical characteristics of the printed traces, such as nozzle/cartridge and sintering temperatures, drop spacing, printing thickness, and sintering duration [31]. Kim et al. [31] presented thorough experimental characterization of the electrical properties as well as the uniformity of inkjet-printed silver lines. Inkjet nozzle temperature, platen temperature, drop spacing, number of printed layers, and sintering temperature, as well as duration, were the major printing parameters that were investigated. The effects of these factors were solely investigated, as the study did not result in any optimal parameters to be recommended.

In addition, the effect of these parameters is limited to the electrical characteristics of the printed traces, while the geometry of the printed patterns, which has several variables of interest, significantly controls the electromechanical properties of the stretchable circuits [23,27]. Abu-Khalaf et al. [27] experimentally characterized inkjet-printed serpent shapes, namely horseshoe and sinusoidal shapes in terms of the geometry effect on the breakdown strain. They variated the number of cycles, the amplitude and the line width of the sinusoidal shapes. On the other hand, only the angle was variated in the case of the horseshoe pattern. Although, the study reported some experimentally-estimated optimal values, they were not based on optimization techniques, and hence the actual optimal values may lay in between the examined values.

With the increase in energy need worldwide [32], industries are continuously researching enhancements to their energy utilization and processes execution [33,34]. Other studies have investigated various operating parameters for optimal operation [35], either for high scale power demand [36] or a lower scale [37]; however, inkjet optimization studies presented in the literature [38,39,40,41] were mainly concerned with optimal process parameters, such as sintering parameters and nozzle settings. For instance, Mypati et al. [38] studied the effect of sintering parameters and the number of layers on the electrical resistivity of inkjet-printed silver nanoparticle films. They found the optimal parameters using central composite design of experiment (DOE) by minimizing the electrical resistance. Sipilä et al. [39] investigated the optimal printing and sintering parameters of inkjet printing of both silver and copper nanoparticle inks. Moreover, Fauzia et al. [40] investigated optimal inkjet printing parameters, such as waveform settings, pulse voltages, and drop spacing in order to obtain droplets of adequate quality for organic solar cells. 

In this study, the focus is shifted towards evaluating the optimal geometry parameters of inkjet-printed silver nanoparticle (NP) traces on polydimethylsiloxane (PDMS) substrates. Moreover, response surface methodology (RSM) based on DOE is performed while taking into consideration two factors: line width (W) and the number of layers (NL) of straight lines. The optimization process is based on maximizing the breakdown strain while minimizing ink cost and maximizing the gauge factor. Subsequently, the optimal parameters from the straight line analysis (line width and number of layers) are used in the optimization of the horseshoe geometry parameters, namely the number of cycles (NC) and the amplitude (A).

## 2. Materials and Methods 

### 2.1. Substrate Preparation

PDMS (Sylgard184, The Dow Chemical Company, Midland, MI, USA), is a clear silicone elastomer that is prepared by mixing two parts: a curing agent and an elastomer base (1:10 volumetric ratio). The mixture has a viscosity of 3500 cP and a thermal conductivity of 0.27 W/m k, according to the technical data sheet [27]. PDMS is physically and chemically stable, highly stretchable, and biocompatible. It requires a relatively low curing temperature, and its elasticity changes slightly with temperature and time. In addition, it is optically transparent, non-toxic, non-flammable, and chemically inert [22,23,42,43,44]. Because of the aforementioned advantages, PDMS is commonly used as a substrate for inkjet-printed traces. To prepare PDMS substrates, the liquid mixture needs to be degassed in a vacuum chamber for 30 min. This insures that any air bubbles formed during mixing are removed. Next, the liquid PDMS is poured into acrylic molds (100 mm × 40 mm) and cured at 70 °C for 2 h. Once the substrates are cured they can be peeled off from the molds and are ready to be used [24,31,45].

### 2.2. PDMS Surface Treatment 

The hydrophobic nature of PDMS inhibits ink pattern formation [25], therefore chemical or physical methods are used to enhance the wettability of PDMS. This step is crucial; as the wettability of the substrate affects the size and stability of the printed traces [14]. O_2_-plasma surface treatment has been widely used to render PDMS hydrophilicity [25]. In this study, plasma etcher (ZEPTO Diener, Germany) was used to improve the hydrophilicity of PDMS substrates to obtain successful patterning of conductive traces [22,24]. Oxygen was selected as the reactive gas and the process conditions were optimized at full power (50 W) for 20 min.

### 2.3. Silver NP Patterning on O_2_-Plasma Treated PDMS

In this study, silver nanoparticle ink (Silverjet DGP-40LT-15C, Sigma-Aldrich, Inc., St. Louis, MO, USA), with a particle size of 180 nm on average, suspended in a solvent (30–35 weight % dispersion in diethyleneglycol monomethyl ether) was used [46]. This ink was selected to design conductive traces on plasma-treated PDMS substrates due to its high electrical conductivity and low curing temperature [31,47].

A Fujifilm Dimatix Material Printer DMP-2831 (Fujifilm Dimatix Material Printer DMP-2831, FUJIFILM Dimatix, Inc., Santa Clara, CA, USA) was utilized to perform all the experiments. This printer uses disposable piezoelectric drop-on-demand print cartridges. Each cartridge has 16 nozzles that are linearly spaced at 254 µm and a capacity of 1.5 mL to minimize waste of costly inks. In addition, it can be easily replaced to enable printing of a series of inks. Pattern creation and jetting resolution are PC-controlled with nominal drop volumes of 1 or 10 pL.

The characteristics of the inkjet-printed conductive traces are affected by various significant printing parameters (e.g., drop spacing, number of printed layers, platen and nozzle temperature). Mainly, these parameters directly affect the conductance, resistance, durability, and dimensions of the printed traces [14,31]. The inkjet printing process parameters were set based on optimal values recommended in [27,31], as shown in Table 1. After the printing concluded, the resulting patterns were cured in a conventional oven (VACUCELL, MMM Medcenter Einrichtungen GmbH, Munich, Germany) for ink sintering purposes. The sintering process is crucial to remove the remaining liquid solvent, which could result in fusing the silver nanoparticles [14,47]. 

### 2.4. Development of Experimental Framework

#### 2.4.1. Background

An experimental design begins by identifying the parameters that influence the outcome of the experiment, this is known as screening. The screening starts by including all controllable factors that may affect the experiment’s results, then excluding the least significant ones. Next, the optimal values for those parameters are evaluated by a process known as optimization. Depending on the desired objectives of the experiment and the number of factors that can be examined, the experimental design is selected [48].

For instance, a Plackett–Burman design is useful if the information provided about the examined system is insufficient or the screening is conducted for a large number of factors. However, if the significant factors are known and one is trying to understand the interactions between them, then a full factorial design is preferred. This design takes into consideration all the possible combinations of all the levels of the factors of interest and it also results in detailed interaction information. In particular, response surface methodology (RSM), which is a multi-full factorial experimental design, utilizes the response to find mathematical equations that describe the relationship between the independent factors and the response [49,50].

#### 2.4.2. Response Surface Methodology 

In this study, response surface methodology (RSM) was used to study the interactions between significant geometrical factors and the electromechanical properties of inkjet-printed stretchable circuits represented by their breakdown strain and gauge factor. The breakdown strain is defined as the maximum strain at which the stretchable circuit loses its electrical conductivity [23]. While the gauge factor (GF) is defined as the relative change in the relative resistance of the printed line to the mechanical strain as shown in Equation (1). The gauge factor represents the sensitivity of the printed circuits where these circuits can be employed as strain gauge sensors. Generally, a conventional strain gauge sensor (with a metal-foil substrate) has a gauge factor between 2 and 5, whereas the gauge factor of a polymer-based strain gauge sensor could reach up to 100 [30].

(1) GF=(R2−R1)R1S

where $R_{2}$ is the resistance of the printed line after applying the strain (Ohm), $R_{1}$ the initial resistance (Ohm), and S the amount of applied mechanical strain (%).

In this study, RSM DOE was performed while taking into consideration two factors: line width and number of layers for the straight line that maximize the breakdown strain and the gauge factor, while minimizing the ink cost, which is calculated from Equation (2). The optimal parameters from the straight line analysis (line width and number of layers) were used in the optimization of the horseshoe pattern parameters: the number of cycles and the amplitude. We chose to neglect the effect of nozzle and platen temperatures and set them to the constant values in Table 1. This is based on the observation that silver NP ink is stable, and therefore there is no need to vary the nozzle temperature. Furthermore, heating the substrate at temperatures other than optimal would result in films that either dry too fast, as the solvent dries too quickly, leaving behind gaps, or too slow, where the material is given time to crystallize out of the solvent without forming a uniform film. In the case of printing multiple layers, the temperature of the platen was set at 60 °C in order to allow the sintering of the layers one by one without affecting the line width [31,51] and as recommended by [38]. Moreover, in order to control the line width and based on [27], the drop spacing was set to be constant at 30 microns.

(2) $$C_{PT}=C_{SI}\times V_{I}\times \rho_{I}$$

where $C_{PT}$ is the cost of printed traces (Euro),$\;C_{SI}$ ink cost (Euro/g), $V_{I}$ volume of deposited ink (L) and $\rho_{I}$ is the silver nanoparticle ink density (g/L). $C_{SI}\;$equals 19.72 Euro, while $\rho_{I}$ equals 1.45 g/L [46]. $V_{I}$ was estimated as follows;

(3) $$V_{I}=N_{BP}\times J_{R}\times 10^{-12}$$

where $N_{BP}$ is the number of black pixels in an image of the pattern as the nozzles fire once at each black pixel, while $J_{R}$ is the jetting resolution (pL). Note that the number of black pixels was found using a MATLAB code.

#### 2.4.3. Pattern Design: Straight Line and Horseshoe 

**Figure 1:**
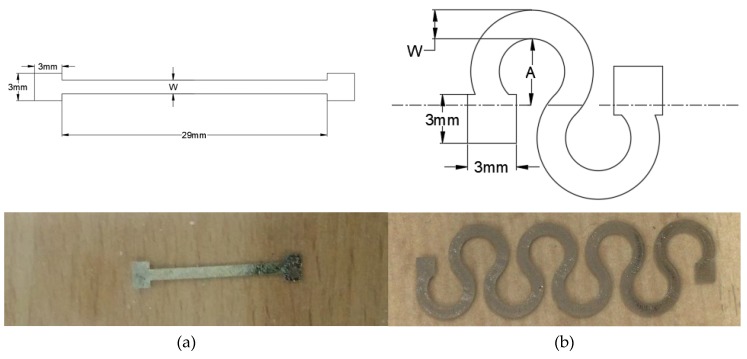
The geometry parameters of: (**a**) straight line and (**b**) horseshoe pattern used in the RSM DOE.

The optimal geometry parameters of straight line and horseshoe patterns were found using RSM DOE, where the optimal parameters from the straight line were used as fixed inputs into the optimization process of the horseshoe patterns. For straight line traces, the number of layers and the line width were the parameters to be optimized. While for the horseshoe pattern, the parameters were the number of cycles and amplitude as shown in Figure 1. The selection of the trial parameters was based on the preliminary experimental results in [27]. Table 2 illustrates the geometry parameters of the horseshoe and the straight line patterns used in the RSM DOE. It should be noted that, due to the dimensions of the PDMS substrate, the number of cycles was limited to four. Moreover, each pattern was printed at least twice in order to confirm the repeatability of the results, which was inspected using analysis of variance (ANOVA), where SPSS 20 (IBM, Armonk, NY, USA) was used for this purpose. If the *p*-value from the ANOVA test is larger than a significance level of 0.05, then the null hypothesis is rejected, indicating that a statistical difference between experimental trials does not exist.

The RSM DOE was performed using Minitab 16 statistical software (Minitab Inc., State College, PA, USA). For straight line traces, the design consists of more than 60 experiments with at least two trials at each combination of parameter levels. On the other hand, the design for the horseshoe pattern consists of 18 experiments with at least two trials at each combination of parameter levels. The DOE was run at an α value of 0.05, which is equivalent to a 95% confidence level.

## 3. Results and Discussion

### 3.1. Data Analysis 

#### 3.1.1. Straight Line

As previously mentioned, it is important to evaluate the statistical significance of the breakdown strain, ink cost, and gauge factor between the experimental trials. The ANOVA test concluded that there was no statistical significance between the experimental trials where the *p*-values were larger than the significance level (0.05) and so the null hypothesis was rejected. The estimation of the breakdown strain using the geometry parameters is vital in order to determine the optimal parameters to be used based on the desired application. This could be limited to the circuit’s dimensions or the amount of required maximum breakdown strain. Table 3 shows the estimated correlation parameters of the breakdown strain, ink cost, and gauge factor. As it can be seen, the factors: line width and number of layers, were significant in regards to the response variables (breakdown strain, ink cost, and gauge factor) with very low *p*-values. Additionally, the two-way interaction components were found to be significant.

Moreover, the results in Table 3 indicate that the square of the line width has a more significant effect on the breakdown strain compared to the line width alone. Increasing the line width gives the printed pattern a higher ability to endure strain. Specifically, as the width increases (more nanoparticles), the possibility of the particles dispatching from each other decreases (larger area) and accordingly the line maintains its electrical conductivity. On the other hand, increasing the number of layers increases the amount of bulk material that will ease the formulation of cracks in the printed patterns. The gauge factor, which is related to the change in the resistance and the amount of breakdown strain, increases as the resistance (R) decreases, which in return depends on the number of layers and line width as indicated in Equation (4). In particular, these parameters control the cross sectional area of the line. From the *p*-values, it could be noted that the effect of the line width on the gauge factor is smaller than the number of layers.

(4) $$R=\frac{\rho \times L}{A}$$

where ρ is the electrical resistivity of the conductive ink (Ohm.m), L is the length of the conductive line (mm), A is the cross-sectional area of the line (mm^2^). 

Additionally, the regression coefficient of the GF was low due to significant variations in the pattern’s resistance, even at the same process conditions. This could be explained by the fact that the resistance of the traces is highly dependent on the dispersion homogeneity of the silver nanoparticles in the ink, where the silver nanoparticles could aggregate with time and disturb the dispersion homogeneity. The latter affects the deposition of uniform amounts of particles in the pattern, which in turn affects the resistance of the printed traces.

**Figure 2:**
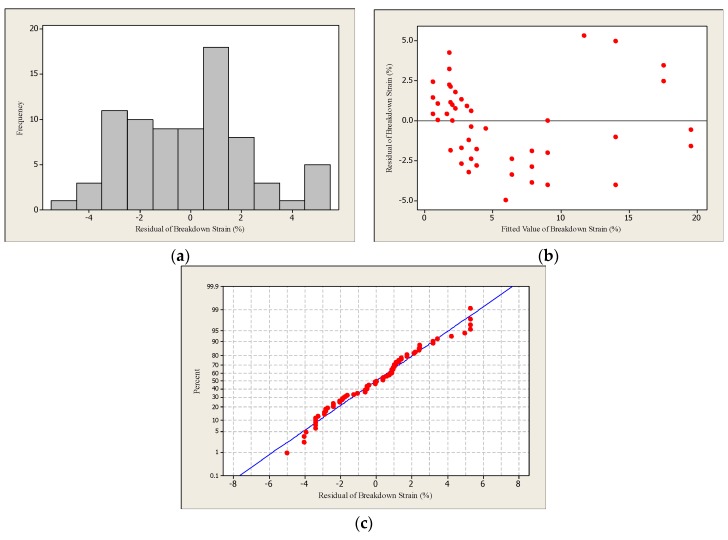
The residual plots: (**a**) histogram, (**b**) residuals vs. fits and (**c**) normal probability of the breakdown strain regression model of the straight line traces.

The residual plots play an important role in finding the outliers (extreme values) in the observations, where these plots could provide a more detailed conclusion compared with box plots. Furthermore, these plots are used to evaluate the goodness of fit of the developed regression model. Figure 2 demonstrates the residual plots of the breakdown strain of the straight line regression model, while Table 4 presents the interpretations of the residual plots.

#### 3.1.2. Horseshoe 

Similar to the straight line, the ANOVA test was used to assess the statistical significance between the experimental trials of the horseshoe patterns, where the results indicate that there is no statistical significance between the experimental trials. Afterwards, the correlation that describes the effect of the amplitude and number of cycles on the breakdown strain, ink cost, and gauge factor was found using RSM DOE. Table 5 shows the estimated correlation parameters of the breakdown strain, ink cost, and gauge factor. 

It can be depicted from Table 5 that the breakdown strain of the horseshoe pattern is highly affected by the amplitude and number of cycles, as well as the interaction between them, with *p*-values < 0.01, while there was no statistical significance for the square of these parameters. The breakdown strain coefficient demonstrates that increasing the number of cycles and the wave amplitude increases the surface area that is exposed to the force and increases the stress applied on the traces, which in return, decreases the ability of the pattern to sustain strain. It can be noted that only the constant, number of cycles, and the interaction between the number of cycles and the amplitude have significant statistical significance on the gauge factor, where increasing the number of cycles increases the sensitivity of the pattern to the strain.

**Figure 3:**
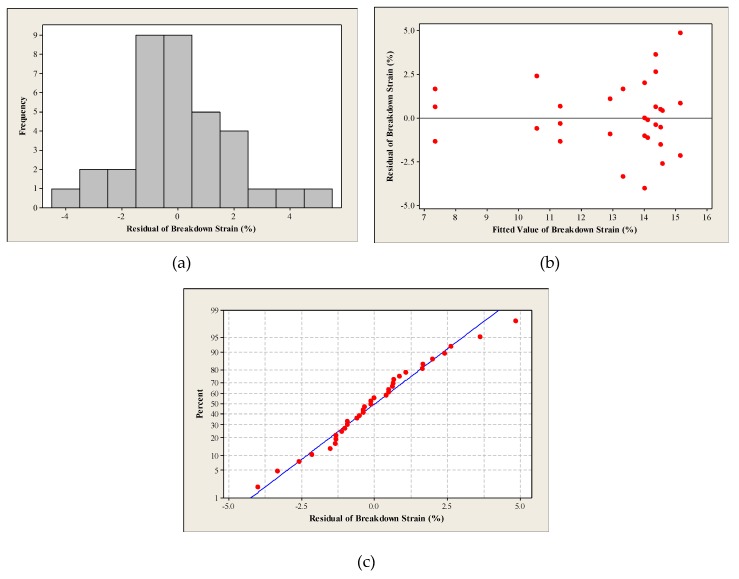
The residual plots: (**a**) histogram, (**b**) residuals vs. fits and (**c**) normal probability of the breakdown strain regression model of the horseshoe pattern.

The regression coefficients of the correlations (breakdown strain and GF) for the horseshoe pattern were lower than those for the straight line, where the deposition of the serpent shape using inkjet printing is more complex compared to printing straight lines. Hence, uncontrolled variables, such as the dispersion homogeneity of the silver nanoparticles in the ink, have a larger impact on the breakdown strain and the resistance of the traces. Similar to the straight line, the residual plots were used to evaluate the goodness of fit, as well as to examine the outliers. Figure 3 shows the residual plots of the breakdown strain of the horseshoe regression model, while Table 6 presents the interpretations of the residual plots.

### 3.2. Optimization of Geometrical Parameters 

#### 3.2.1. Straight Line

**Figure 4:**
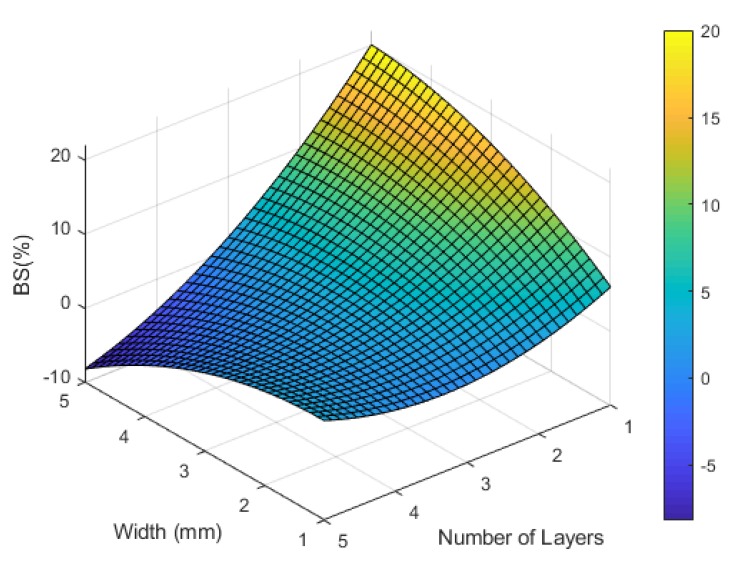
The predicted relationship between the line width, number of layers, and the breakdown strain of a straight line.

**Figure 5:**
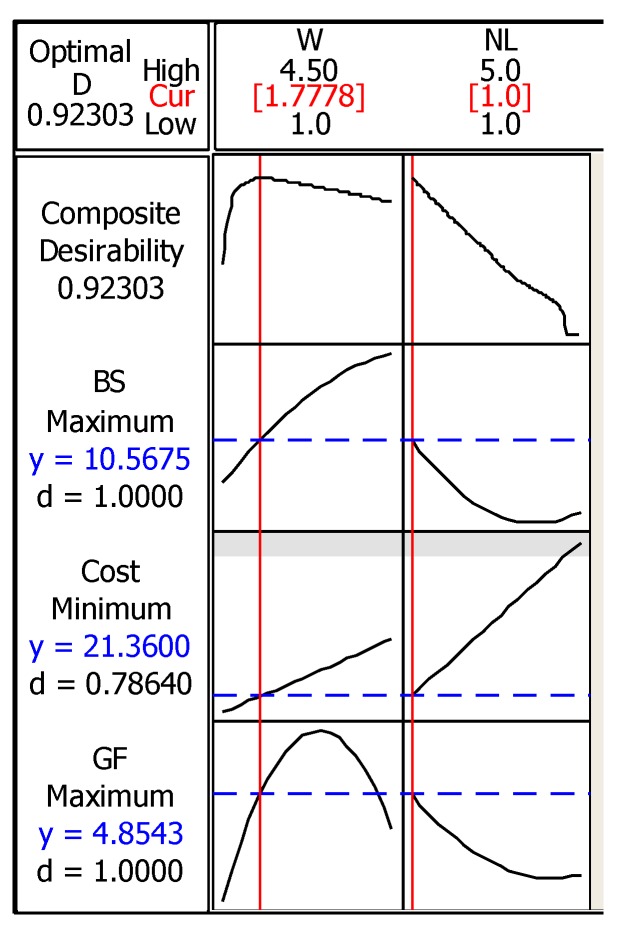
Surface response optimization plot of breakdown strain, ink cost, and gauge factor of the inkjet-printed straight line.

The breakdown strain of the printed circuit increased by increasing the line width as shown in Figure 4. There is no close turning point that results in geometry parameters that fit within the dimensions of the PDMS substrate, and therefore it is essential to highlight one potential application to limit the required maximum breakdown strain. Many vital signs can be measured with wearable and sensitive strain gauge sensors such as respiratory rate and heart rate, where the physiological indications of these signs usually cause a small amount of strain (≤5%) [52,53]. It was reported in [30] that thin-film nanoparticle-based strain gauge sensors usually bear up to 10% strain, therefore a 10% maximum breakdown strain was added to the optimization process. Figure 5 shows the RSM DOE optimization results of the inkjet-printed straight line.

The lowest ink cost in the dataset (12.61 × $10^{-6}$ Euro) was entered as the minimization target of the ink cost, whereas a gauge factor of 2 was used as the maximization target. As it can be seen in Figure 5, the minimization of the cost had the lowest desirability among the other responses, where the composite desirability of the outputs was satisfactory at the optimal line width of 1.78 mm with one printed layer. The predicted breakdown strain of the line at these parameters was 10.57% with an ink cost of 21.36 × $10^{-6}\;$Euro and gauge factor of 4.85. The experimental validation of the obtained results is essential in order to evaluate the accuracy of the developed model before proceeding with these results for the optimization of the horseshoe patterns. Six straight lines with 1.78 mm line width and one printed layer were tested in order to validate the optimization model, the average maximum breakdown strain of these lines was 11.5% with an error of 8.83%.

#### 3.2.2. Horseshoe 

**Figure 6:**
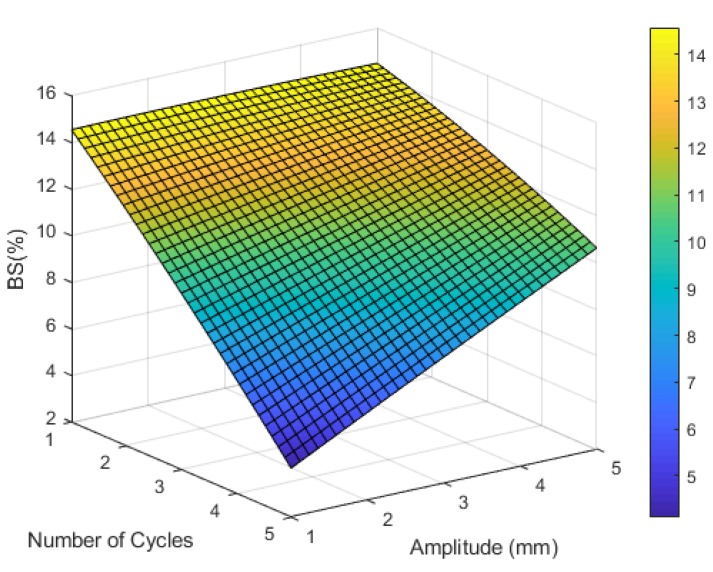
The predicted relationship between the number of cycles, wave amplitude, and the breakdown strain of a horseshoe pattern.

**Figure 7:**
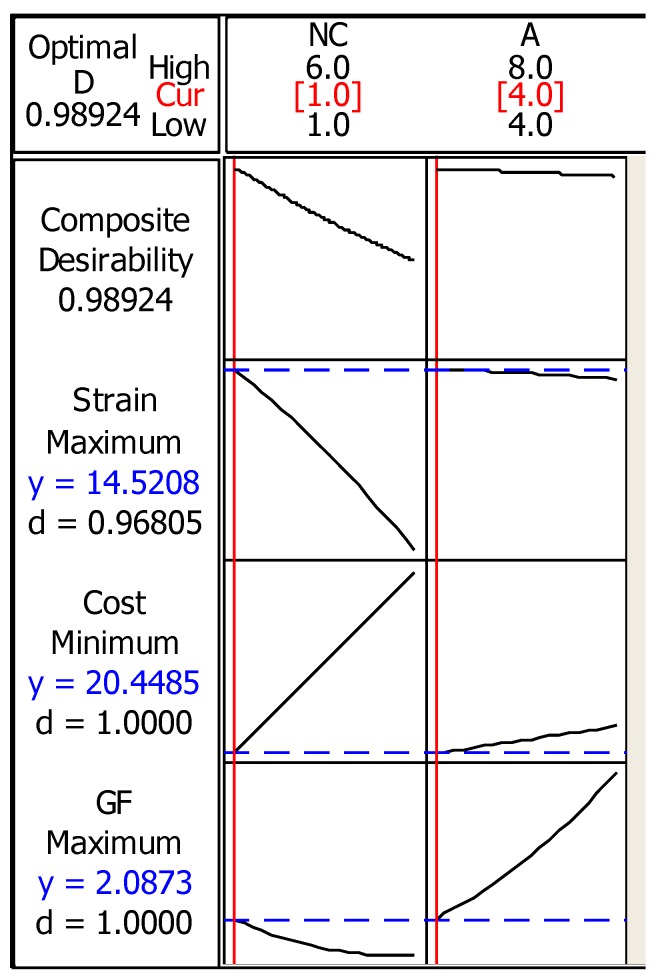
Surface response optimization plot of breakdown strain, ink cost, and gauge factor of the inkjet-printed horseshoe pattern.

The results indicate that there is no significant effect of the amplitude on the breakdown strain, while the number of cycles has an inverse effect on the breakdown strain as shown in Figure 6. The optimal horseshoe geometry parameters that maximize the breakdown strain and GF while minimizing the ink cost were one cycle and 4 mm amplitude at the optimal line width and number of layers (1.78 mm and 1 layer) obtained from the straight line optimization. Figure 7 shows the RSM DOE optimization results of the inkjet-printed horseshoe pattern.

The lowest ink cost in the dataset (20.45 × $10^{-6}$ Euro) was entered as the minimization target of the ink cost, whereas a gauge factor of 2 was used as the maximization target. As demonstrated by Figure 7, the maximization of the breakdown strain had the lowest desirability among the other responses where the composite desirability of the outputs was satisfactory. The predicted breakdown strain of the line with these parameters was 14.52% with an ink cost of 20.45 × $10^{-6}\;$Euro and a gauge factor of 2.09. It can be noted that the horseshoe pattern sustained higher strain with lower ink cost compared to the straight line, however, the gauge factor was smaller. The shape of the horseshoe as reported in the literature [27] plays a major role in distributing the load on the printed traces, which increases the ability of the serpent pattern to sustain more strain compared with straight lines.

### 3.3. Limitation and Future Work

The conductivity and stretchability of the silver inkjet-printed lines are not only affected by the inkjet printing parameters, sintering, and substrate, but are also highly dependent on the amount of silver loaded in the ink, the silver nanoparticle size, used binding agents, and the continuity of the printed lines. In this specific experiment, we expect a resistivity of 20 µΩ.cm for NP silver ink with a particle size of 180 nm [46] and we aim for a desired stretchability of 10% strain. Hence, the results found in this study are transferable to inks of similar properties. If another type of ink is to be tested, the methodology presented in this paper can be replicated to result in the optimal dimensions of the printed patterns [54].

## 4. Conclusions 

This paper presents a comprehensive study of the inkjet printing geometrical parameters that result in optimal silver NPs traces. Specifically, we aimed to print traces with a high strain and gauge factor, and low ink cost, on PDMS substrates. While most studies in the literature have focused on the optimization of printing parameters such as sintering temperatures and drop spacing, the focus was shifted in this study towards the optimization of geometrical parameters. Hence, straight lines and horseshoe patterns were examined. The printing input factors for straight lines were: the number of inkjet-printed layers and the line width, and for horseshoe patterns: the number of cycles and the amplitude. Although, the effect of the aforementioned parameters can be explained using physical relationships to a certain extent, these relationships do not evaluate the significance of each parameter, nor do they predict the effect of varying combinations of these parameters on the desired circuit performance.

Moreover, experimental optimization does not result in precise optimal parameters but rather gives a rough estimate depending on the examined levels of the parameters of interest. Hence, RSM-based DOE was performed, where the results indicated that the optimal parameters for the straight line trace are one layer of silver NPs at a 1.78 mm line width. These parameters yielded up to 10% strain. While the horseshoe pattern, at the optimal number of layers and line width, was optimized at 4 mm amplitude and one cycle, yielding a strain up to 15%. The obtained results were supported by explaining the physical implications behind the observed behavior. It is also worth mentioning that this study can be replicated using other conductive inks, such as gold and copper NPs, as the factors of interest and the desired response remain the same. 

## Figures and Tables

**Table 1:** Printing process parameters.

Printing Parameters	Values
Droplet Speed (m/s)	8
Firing Voltage (V)	23
Nozzle/Cartridge Temperature (°C)	32
Platen Temperature for Single Layer Patterns (°C)	24 (Room Temperature)
Platen Temperature for Multiple layers Patterns (°C)	60
Sintering Temperature (°C)	110
Sintering Time (mins)	60
Jetting Resolution (pL)	10

**Table 2:** The geometry parameters of the horseshoe and the straight line patterns.

Straight Line		Horseshoe	
Parameter	Values	Parameter	Values
Line Width (W)	1,1.5,2,2.5,3.5,4.5	Amplitude (A)	4,6,8
Number of Layers (NL)	1,2,3,4,5	Number of Cycles (NC)	1,2,4

**Table 3:** The estimated values of the correlation parameters of the breakdown strain, ink cost, and gauge factor as well as their *p*-values for the straight line patterns.

Term	Breakdown Strain (%) *		Cost (Euro) **		GF ***	
	Coefficient	*p*-Value	Coefficient	*p*-Value	Coefficient	*p*-Value
Constant	2.90	0	3.75 × $10^{-8}$	0	−9.54	0.309
W (mm)	9.58	0.417	−3.75 × $10^{-7}$	0	12.79	0.027
NL	−5.19	0	1.62 × $10^{-6}$	0	0.295	0.001
W^2^	−0.742	0.081	1.49 × $10^{-7}$	0.019	−1.85	0.003
NL^2^	1.03	0	3.75 × $10^{-8}$	0.266	0.326	0.252
NL × W	−1.61	0.01	1.12 × $10^{-5}$	0	−1.75	0.011

* R^2^ = 82.44%, ** R^2^ = 99.98%, *** R^2^ = 25.94%.

**Table 4:** The interpretation of the residual plots of the breakdown strain of the straight line regression model.

Plot	Results
Histogram	The histogram is roughly bell-shaped which means that the residual error data are normally distributed.
Residuals versus fitted values	The points are randomly scattered around the zero reference which supports the assumption of constant variance.
Normal probability plot	The plot confirms that the data are normally distributed with some outliers. Moreover, the data show strong correlation to one another, resulting in a high Coefficient of Correlation (R = 82.44%).

**Table 5:** The estimated values of the correlation parameters of the breakdown strain, ink cost, and gauge factor, as well as their *p*-values for the horseshoe patterns.

Term	Breakdown Strain (%) *		Cost (Euro) **		GF ***	
	Coefficient	*p*-Value	Coefficient	*p*-Value	Coefficient	*p*-Value
Constant	17.23	0	4.94	0	0.772	0.012
NC	−2.65	0.005	4.51	0	0.397	0.002
A (mm)	−0.362	0.002	−0.111	0	0.148	0.278
NC^2^	−0.0623	0.662	−0.028	0	0.042	0.724
A^2^	−0.0124	0.944	0.016	0.035	0.07	0.633
NC × A	0.413	0.008	2.80	0	−0.21	0.091

* R^2^ = 59.02%, ** R^2^ = 100%, *** R^2^ = 42.36%.

**Table 6:** The interpretation of the residual plots of the breakdown strain of the horseshoe regression model.

Plot	Results
Histogram	The histogram is bell-shaped which means that the residual error data are normally distributed.
Residuals versus fitted values	The points are randomly scattered around the zero reference which supports the assumption of constant variance.
Normal probability plot	The plot confirms that the data are normally distributed with some outliers. Moreover, the data show good correlation to one another, with acceptable Coefficient of Correlation (R = 59.02%).
